# Association between Traditional Chinese Medicine patterns and dry eye in the Chinese population: A cross-sectional study

**DOI:** 10.1097/MD.0000000000046955

**Published:** 2026-01-02

**Authors:** Jing Yu, Yihan Guo, Lei Tian, Xiaoniao Chen, Kai Cao, Lei Zhu, Mengnan Zhao, Lixin Qiu, Ying Jie

**Affiliations:** a Beijing Institute of Ophthalmology, Beijing Tongren Eye Center, Beijing Tongren Hospital, Capital Medical University; Beijing Ophthalmology & Visual Sciences Key Laboratory, Beijing, China; b Department of Ophthalmology, The Third Medical Center of Chinese People’s Liberation Army General Hospital, Beijing, China.

**Keywords:** dry eye, Traditional Chinese Medicine, Traditional Chinese Medicine patterns

## Abstract

We investigated the distribution of traditional Chinese medicine (TCM) patterns and their correlation with dry eye. This cross-sectional study was conducted at Beijing Tongren Hospital’s Dry Eye Diagnosis and Treatment Center and included 205 patients diagnosed with dry eye following the TFOS DEWS II criteria. All patients underwent a comprehensive clinical examination for dry eye and completed the dry eye TCM questionnaire. TCM pattern differentiation was performed for all patients. We investigated the distribution of TCM patterns among patients with dry eye and examined the correlation between TCM patterns and dry eye indicators using Spearman correlation analysis. Based on patients’ symptoms, tongue coating, and pulse conditions, the 205 patients were classified into 11 TCM patterns. The 3 most common TCM patterns were qi stagnation and blood stasis pattern, spleen-kidney yang deficiency pattern, and qi-yin deficiency pattern. There were significant correlations between these TCM patterns and 1, 3, and 4 dry eye indicators, respectively. The proportion of TCM patterns and the correlations between TCM patterns and dry eye indicators varied among different dry eye subtypes. We preliminary investigated the distribution of TCM patterns among patients with dry eye and found a certain correlation between TCM patterns and dry eye indicators. These findings provide a theoretical basis for further research on TCM for treating dry eye.

## 1. Introduction

Dry eye disease (DED) is a chronic ocular surface disease caused by several factors. DED is characterized by tear film instability or imbalance in the ocular surface microenvironment due to abnormalities in the quality, quantity, and dynamics of tears. It may be accompanied by ocular surface inflammation, tissue damage, and neurological abnormalities, leading to various symptoms and/or visual impairments.^[1]^ With its increasing prevalence, DED has become one of the most common ocular surface diseases that affect people’s quality of life. According to the second edition of the International Dry Eye Workshop (DEWS) II consensus,^[2]^ DED can be classified into aqueous deficiency, evaporative, and mixed types based on its etiology. As described in the report, the boundaries between aqueous deficiency DED and evaporative DED are not clearly defined, and both are associated with several factors.^[3]^

In Traditional Chinese Medicine (TCM), DED can be categorized as “Bai Se Zheng” (white and astringent syndrome) and “Shen Shui Jiang Ku” (essence-wasting due to water depletion), characterized by dry and gritty sensation of the eyes, blurred vision, and the absence of redness or the presence of fine red veins on the sclera.^[4]^ According to TCM theory, lingering pathogenic heat, deficiency of Qi and Yin, insufficiency of the liver and kidneys, phlegm-dampness, and blood stasis are involved in the pathogenesis of DED. They impair the ability of Qi, blood, and body fluids to nourish the eyes, resulting in impaired eye nourishment.^[5]^ According to TCM theory, the essential qi of the 5 zang organs and 6 fu organs ascends to nourish the eyes, with tears being its external manifestation. Furthermore, the eyes are closely connected to the 5 zang organs, and this qi circulates around the eyes through the Five Wheels.^[4]^ These records fully demonstrate the close relationship between DED and various organs.

TCM has shown certain advantages in improving the symptoms and signs of patients with DED.^[6–8]^ However, there is currently no unified standard for treating DED with TCM, and different TCM practitioners diagnose and treat DED based on their own experience. The diagnostic criteria for TCM patterns often remain at the macroscopic level. Pattern differentiation and treatment are fundamental principles in TCM for understanding and treating diseases, representing a special research and treatment approach in TCM.^[4]^ Many practitioners classify DED into various patterns based on the principles of pattern differentiation and treat each pattern accordingly. In this study, we primarily investigated the distribution of TCM patterns in DED at the microscopic level and explored their respective relationships with the symptoms and signs of DED. In addition, we measured the correlation between TCM patterns and diagnostic indicators of DED in Western medicine. Combining the TCM concepts of “holistic view” and “prevention of disease,” this study aimed to provide a reference for early intervention and treatment of DED.

## 2. Materials and methods

### 2.1. General information

**Figure 1.**
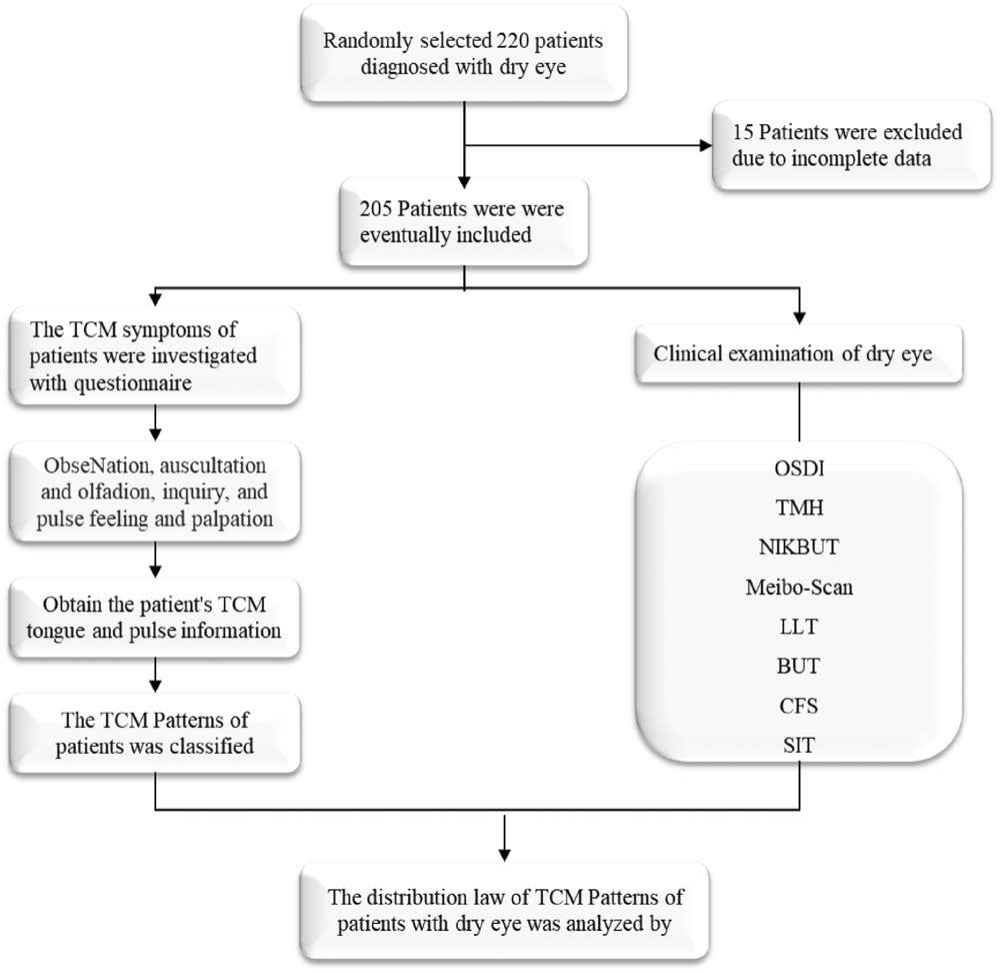
The flow diagram of this study.

This cross-sectional study was conducted at the Dry Eye Diagnosis and Treatment Center of Beijing Tongren Hospital. We randomly included 205 patients diagnosed with DED between January 2022 and November 2024 (52 males and 153 females). All participants were diagnosed following the TFOS DEWS II diagnostic criteria. To avoid potential interference from the correlation between both eyes of the same patient, we selected the eye with more severe symptoms for analysis. A comprehensive DED diagnosis was performed for all patients, including the completion of the “Traditional Chinese Medicine Questionnaire for Dry Eye” (Fig. 1).

Patients were divided into the following groups based on their age (each group spanning a decade): 20 to 30 years, 31 to 40 years, 41 to 50 years, 51 to 60 years, and > 60 years. Inclusion criteria were as follows: meeting the TFOS DEWS II diagnostic criteria for DED, providing written informed consent, and being able to complete the “Traditional Chinese Medicine Questionnaire for Dry Eye.” Exclusion criteria were as follows: age below 18 or above 75 years, presence of severe cardiovascular or cerebrovascular diseases or mental disorders, noncompliance with examinations, a history of ocular surgery or chemical burns, and a history of long-term ocular medication.

This study was conducted following the Helsinki principles and was approved by the Ethics Committee of Beijing Tongren Hospital, Capital Medical University.

### 2.2. Diagnostic criteria and process

Based on the clinical examination, all participants underwent the following assessments in the specified order: Before any examinations, participants completed the ocular surface disease index (OSDI) questionnaire. We used the ocular surface analyzer (Oculus, Wetzlar, Germany) to assess tear meniscus height (TMH), noninvasive tear film breakup time (NIKBUT), and meibomian gland imaging. The LipiView interferometer (TearScience, Morrisville) was used to measure tear film lipid layer thickness (LLT), incomplete blinks, and total blinks. Thereafter, 1% to 2% fluorescein sodium dye was instilled into the lower conjunctival sac of the participants, and corneal fluorescein staining (CFS) score was assessed. After a 30-minute rest period, the Schirmer I test (without anesthesia) (SIT) was performed for 5 minutes.

Based on the DED indicators, the diagnosis was made following the TFOS DEWS II criteria. This criteria includes the following: (1) Symptoms: OSDI questionnaire score ≥ 13 or Dry Eye Severity Scale score ≥ 6. (2) At least one positive result from the following tests: tear film breakup time (FBUT) ≤ 10s, inter-eye tear osmolarity difference > 8 mOsm/L, corneal staining with > 5 spots, conjunctival staining with > 9 spots, or lid margin staining (≥2 mm length or ≥ 25% width). Based on the DED classification criteria, patients were divided into the following groups: mixed-type DED, evaporative-type DED, and aqueous-deficient DED. All examinations were performed by the same physician, in the same examination room, and under consistent conditions.

### 2.3. TCM assessment

The “Traditional Chinese Medicine Questionnaire for Dry Eye” was employed for assessment, comprising 50 commonly observed TCM symptoms associated with DED. This questionnaire was rigorously developed through a comprehensive literature review and expert clinical consensus. Its design drew upon *The Criteria of Diagnosis and Therapeutic Effects of Diseases and Syndromes in Traditional Chinese Medicine*, *Guidelines for Clinical Research of New Chinese Medicines*, and the internationally recognized *TFOS DEWS II Report* on dry eye diagnosis.^[9–12]^ All assessments were conducted and recorded by a single experienced TCM practitioner to ensure consistency.

Clinical data were collected and organized for each patient who met the inclusion criteria. Based on the principles of TCM, including the differentiation of organs and meridians, the 8 principles, and the differentiation of qi, blood, yin, and yang, patients were classified into different TCM patterns based on their symptoms.

### 2.4. Statistical analysis

We analyzed data using SPSS (version 26.0). The sample size calculation was per the following formula: n = Z^2^_α/2_ ·P(1 − P)/E^2^. The incidence rate of dry eye is: *P* = 33.7%, and the expected error is: E = 0.1. Consequently, a minimum enrollment of 86 participants is needed. Quantitative data are expressed as mean ± standard deviation. The Shapiro–Wilk test was employed to assess whether the measured indicators followed a normal distribution. Spearman correlation analysis was utilized to assess the correlation between different TCM patterns and DED subtypes and measure the correlation between TCM patterns and DED indicators. Furthermore, subgroup analyses were conducted using Spearman correlation analysis: examining the correlation between TCM patterns and DED indicators across different subtypes of DED, and measuring the correlation between TCM symptoms and DED indicators across different TCM patterns. A correlation coefficient (*r*) > 0 indicated a positive correlation between 2 variables, while *R* < 0 indicated a negative correlation. Statistical tests were 2-sided, and *P* < .05 was considered statistically significant.

## 3. Results

### 3.1. General information

#### 3.1.1. Sample size and age

**Figure 2.**
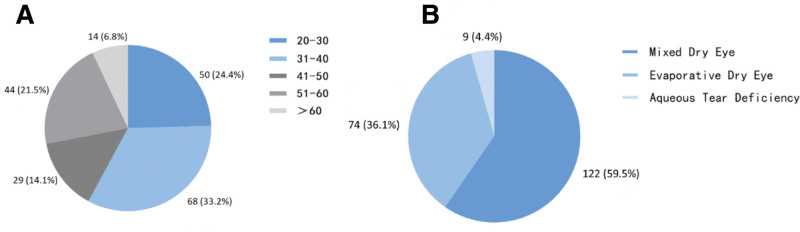
Patient clinical characteristics.

In total, 205 patients and 205 eyes were included in this study. The average age of the patients was 40.43 ± 13.12 years, ranging from 20 to 73 years. Among them, 153 were female (74.63%) and 52 were male (25.37%). The patients were grouped based on their age, with each group spanning a 10-year range: 20 to 30 years, 31 to 40 years, 41 to 50 years, 51 to 60 years, and > 60 years (Fig. 2). Among these groups, more patients were in the 31 to 40 age group (68 cases, 33.17%).

#### 3.1.2. Duration of disease

In this survey, the patients had a maximum disease duration of 30 years and a minimum disease duration of only 6 months. The average duration of the disease was 34.89 ± 31.33 months. The disease course in each age group is shown in Table 1.

**Table 1:** Disease course by age group.

Patients’ age (years)	Disease course (month)
20–30	27.11 ± 28.14
31–40	26.11 ± 21.95
41–50	43.63 ± 36.30
51–60	44.14 ± 32.99
>60	61.00 ± 45.55

### 3.2. DED subtypes

Patients with DED were classified following the TFOS DEWS II classification. The proportion of each DED type was as follows: 122 cases (59.51%) of mixed type, 74 cases (36.10%) of evaporative type, and 9 cases (4.39%) of aqueous-deficient type (Fig. 2).

### 3.3. TCM patterns

#### 3.3.1. Distribution of TCM patterns

According to TCM diagnostic methods, combined with patients’ symptoms, tongue coating, and pulse condition, we divided the 205 patients into 11 groups of TCM patterns. From the highest to the lowest, the distribution of these patterns was as follows: Qi stagnation and blood stasis; spleen and kidney yang deficiency; Qi and Yin deficiency; liver and kidney yin deficiency; liver depression and Qi stagnation; cold congealing and blood stasis; Qi and blood deficiency; spleen deficiency with dampness accumulation; Qi deficiency and blood stasis; heart, liver, and blood deficiency; and liver and gallbladder damp-heat.

Among the 122 patients with mixed-type DED, the most common TCM patterns were as follows: Qi stagnation and blood stasis; spleen and kidney yang deficiency; Qi and Yin deficiency; and liver and kidney yin deficiency. Among the 74 patients with evaporative DED, the most common TCM patterns were as follows: Qi stagnation and blood stasis; spleen and kidney yang deficiency; Qi and Yin deficiency; and liver and kidney yin deficiency. Among the 9 patients with aqueous-deficient DED, the most common TCM patterns were as follows: Qi stagnation and blood stasis; cold congealing and blood stasis; and Qi and Yin deficiency (Table 2). We conducted a correlation analysis between DED subtypes and TCM patterns. The results showed a significant correlation between cold congealing and blood stasis pattern and aqueous-deficient DED (*r* = 0.232, *P* = .013) (Table 3).

**Table 2:** Distribution of TCM patterns among patients with DED.

TCM pattern	Dry eye classification n (%)			Total n (%)
	Mixed dry eye (N = 122)	Evaporative dry eye (N = 74)	Aqueous tear deficiency (N = 9)	
Liver and kidney yin deficiency	13 (10.66)	7 (9.46)	0 (0.00)	20 (9.76)
Deficiency of spleen and kidney yang	24 (19.67)	18 (24.32)	0 (0.00)	42 (20.49)
Cold coagulation blood stasis	2 (1.64)	2 (2.70)	2 (22.22)	6 (2.93)
Deficiency of Qi and Yin	22 (18.03)	16 (21.62)	2 (22.22)	40 (19.51)
Qi stagnation and blood stasis	39 (31.97)	23 (31.08)	5 (55.56)	67 (32.68)
Spleen deficiency and dampness	2(1.64)	2 (2.70)	0 (0.00)	4 (1.95)
Liver depression and qi stagnation	8 (6.56)	4 (5.41)	0 (0.00)	12 (5.85)
Qi and blood deficiency	4 (3.28)	2 (2.70)	0 (0.00)	6 (2.93)
Qi deficiency and blood stasis	4 (3.28)	0 (0.00)	0 (0.00)	4 (1.75)
Deficiency of heart and liver blood	2 (1.64)	0 (0.00)	0 (0.00)	2 (0.88)
Liver and gallbladder damp heat	2 (1.64)	0 (0.00)	0 (0.00)	2 (0.88)

**Table 3:** Correlation analysis between DED subtypes and TCM patterns.

	Mixed dry eye		Evaporative dry eye		Aqueous tear deficiency	
	*r*	*P*	*r*	*P*	*r*	*P*
Liver and kidney yin deficiency	0.027	.779	0.003	.977	−0.070	.459
Deficiency of spleen and kidney yang	−0.032	.735	0.079	.405	−0.108	.254
Deficiency of Qi and Yin	−0.032	.735	0.033	.726	−0.001	.992
Qi stagnation and blood stasis	−0.025	.789	−0.026	.785	0.121	.199
Cold coagulation blood stasis	−0.088	.351	−0.009	.924	0.232	**.013**
Spleen deficiency and dampness	−0.026	.781	0.039	.680	−0.029	.762
Liver depression and qi stagnation	0.034	.722	−0.013	.891	−0.050	.594
Qi and blood deficiency	0.024	.804	−0.009	.924	−0.035	.710
Qi deficiency and blood stasis	0.110	.244	−0.100	.289	−0.029	.762
Deficiency of heart and liver blood	0.077	.413	−0.071	.456	−0.020	.832
Liver and gallbladder damp heat	0.077	.413	−0.071	.456	−0.020	.832

#### 3.3.2. Relationship between TCM patterns and DED indicators

We analyzed the correlation between TCM patterns and DED indicators. Patients with Qi stagnation and blood stasis pattern had lower NIKBUT grades, and there was a significant negative correlation between them (*r* = −0.186, *P* = .047). Patients with spleen and kidney yang deficiency pattern had lower overall OSDI scores, visual function scores, and environmental trigger factor scores, showing a significant negative correlation (*r* = −0.347, *P* < .001; *r* = −0.234, *P* = .018; and *r* = −0.330, *P* = .001). Patients with Qi and Yin deficiency pattern had shorter first breakup time and shorter average breakup time, showing a significant negative correlation (*r* = −0.214, *P* = .022; and *r* = −0.189, *P* = .044). They also had higher NIKBUT grades and larger SIT values, showing a significant positive correlation (*r* = 0.267, *P* = .004; and *r* = −0.202, *P* = .033) (Table 4).

**Table 4:** Correlation analysis between TCM pattern types and DED indexes.

	Qi stagnation and blood stasis		Deficiency of spleen and kidney yang		Deficiency of Qi and Yin		Liver and kidney yin deficiency		Other	
	*r*	*P*	*r*	*P*	*r*	*P*	*r*	*P*	*r*	*P*
TMH	0.037	.693	0.113	.229	−0.150	.112	0.150	.112	−0.169	.073
NIKBUT										
Time to first rupture	0.111	.242	0.050	.594	−0.214	**.022**	−0.061	.516	0.077	.415
Mean rupture time	0.070	.459	0.159	.090	−0.189	**.044**	−0.115	.224	0.039	.682
Grading	−0.186	**.047**	−0.171	.070	0.267	**.004**	0.106	.260	0.023	.810
LLT	−0.012	.903	−0.012	.898	−0.039	.683	0.023	.811	0.058	.544
Schirmer (no surface hemp)	−0.031	.744	0.011	.908	0.202	**.033**	−0.039	.684	−0.131	.167
OSDI score										
OSDI Total Score	0.170	.087	−0.347	**<.001**	0.120	.229	0.025	.799	0.034	.731
Eye symptoms	0.073	.463	−0.192	.054	0.079	.431	0.045	.655	0.011	.910
Visual function	0.154	.123	−0.234	**.018**	0.080	.422	−0.069	.492	0.020	.844
Environmental factor	0.167	.094	−0.330	**.001**	0.184	.064	0.066	.512	−0.071	.475
Image analysis of meibomian glands										
Upper eyelid	−0.121	.198	0.069	.463	0.055	.562	0.112	.236	−0.048	.610
Lower eyelid	−0.116	.218	0.147	.118	0.026	.786	0.100	.290	−0.105	.265
CFS	−0.036	.706	−0.095	.318	−0.080	.399	0.125	.190	0.162	.087

CFS *=* corneal fluorescein staining, LLT *=* lipid layer thickness, NIKBUT *=* noninvasive tear breakup time**,** OSDI ***=*** Ocular Surface Disease Index, TMH *=* tear meniscus height.

Further analysis was conducted to measure the correlation between TCM patterns and DED indicators among different subtypes of DED patients. Among the 122 patients with mixed-type DED, those with spleen and kidney yang deficiency pattern had significantly lower overall OSDI scores and environmental factor scores, showing a significant negative correlation (*r* = −0.274, *P* = .030; *r* = −0.276, *P* = .030). Patients with Qi and Yin deficiency pattern had higher NIKBUT grades and greater tear secretion values (Schirmer without anesthesia), showing a significant positive correlation (*r* = 0.248, *P* = .042; and *r* = 0.342, *P* = .005). Among the 74 patients with evaporative-type DED, those with Qi stagnation and blood stasis pattern had lower NIKBUT grades, showing a significant negative correlation (*r* = −0.318, *P* = .043). Patients with spleen and kidney yang deficiency pattern had significantly lower overall OSDI scores, visual function scores, and environmental factor scores, showing a significant negative correlation (*r* = −0.445, *P* = .007; *r* = −0.426, *P* = .011; and *r* = −0.397, *P* = .018). Patients with Qi and Yin deficiency pattern had shorter first breakup time and average breakup time of NIKBUT, showing a significant negative correlation. In contrast, NIKBUT grades showed a significant positive correlation with Qi and Yin deficiency pattern (*r* = −0.329, *P* = .036; *r* = −0.324, *P* = .039; and *r* = 0.334, *P* = .033). Patients with liver and kidney yin deficiency pattern had larger values in meibomian gland analysis (lower eyelid), showing a significant positive correlation (*r* = 0.313, *P* = .047) (Table 5).

**Table 5:** The correlation between TCM patterns and DED index based on the DED classification.

		Mixed dry eye					Evaporative dry eye					Aqueous tear deficiency		
		Qi stagnation and blood stasis	Deficiency of spleen and kidney yang	Deficiency of Qi and Yin	Liver and kidney yin deficiency	other	Qi stagnation and blood stasis	Deficiency of spleen and kidney yang	Deficiency of Qi and Yin	Liver and kidney yin deficiency	Other	Qi stagnation and blood stasis	Deficiency of Qi and Yin	Other
TMH	*r*	0.027	0.140	−0.225	0.145	−0.123	0.024	0.137	−0.142	0.226	−0.240	< 0.001	0.354	−0.354
	*P*	.825	.254	.065	.240	.317	.879	.392	.375	.155	.131	1.000	.559	.559
NIKBUT - first burst time	*r*	0.044	0.061	−0.151	−0.041	0.047	0.140	0.086	−0.329*	−0.087	0.183	0.289	0.354	−0.707
	*P*	.721	.621	.220	.742	.702	.384	.591	**.036**	.589	.253	.638	.559	.182
NIKBUT-mean time to rupture	*r*	−0.010	0.157	−0.140	−0.094	0.072	0.146	0.235	−0.324*	−0.146	0.025	−0.289	0.354	< 0.001
	*P*	.938	.200	.255	.447	.561	.362	0.139	**.039**	.363	.876	.638	.559	1.000
grading	*r*	−0.083	−0.201	0.248*	0.038	−0.014	−0.318*	−0.186	0.334*	0.189	0.102			
	*P*	.500	.100	**.042**	.756	.912	**.043**	0.244	**.033**	.237	.525			
LLT	*r*	−0.145	0.157	−0.045	−0.047	0.100	0.218	−0.301	−0.005	0.169	−0.083	−0.577	< 0.001	0.707
	*P*	.240	.202	.716	.704	.419	.182	0.063	.974	.302	.616	.308	1.000	.182
Lacrimal secretion test (Schirmer test)	*r*	0.001	−0.098	0.342**	−0.108	−0.126	−0.028	0.020	0.046	0.032	−0.073	< 0.001	0.354	−0.354
	*P*	.995	.434	**.005**	.387	.314	.860	.904	.774	.841	.649	1.000	.559	.559
OSDI total score	*r*	0.140	−0.274*	0.116	0.057	−0.002	0.149	−0.445**	0.124	−0.010	0.213	0.289	0.354	−0.707
	*P*	.274	**.030**	.363	.660	.986	.392	**.007**	.479	.954	.218	.638	.559	.182
Ocular symptoms	*r*	0.063	−0.123	0.060	0.003	0.015	0.117	−0.317	0.011	0.122	0.120	< 0.001	0.707	−0.707
	*P*	.628	.340	.643	.981	.909	.502	.063	.952	.487	.491	1.000	.182	.182
Visual function	*r*	0.108	−0.142	0.065	−0.043	−0.026	0.164	−0.426*	0.099	−0.096	0.272	0.296	0.363	−0.725
	*P*	.405	.271	.613	.741	.842	.345	**.011**	.570	.582	.114	.628	.548	.165
Environmental factor	*r*	0.113	−0.276*	0.230	0.169	−0.164	0.157	−0.397*	0.187	−0.036	0.081	0.761	−0.745	−0.186
	*P*	.382	**.030**	.072	.190	.203	.366	**.018**	.283	.838	.643	.135	.148	.764
Meibomian gland image analysis - Upper eyelid	*r*	−0.108	0.123	0.006	0.080	−0.029	−0.159	−0.013	0.096	0.172	−0.035	< 0.001	0.395	−0.395
	*P*	.380	.316	.959	.517	.813	.322	.935	.550	.281	.829	1.000	.510	.510
Meibomian gland image analysis - lower eyelid	*r*	−0.120	0.214	−0.048	< 0.001	< 0.001	−0.199	0.108	0.075	0.313*	−0.236	< 0.001	0.559	−0.559
	*P*	.330	.080	.700	1.000	1.000	.211	.501	.642	.047	.137	1.000	.327	.327
CFS	*r*	−0.170	0.035	0.028	0.116	0.103	0.115	−0.266	−0.126	0.175	0.179	< 0.001	−0.707	0.707
	*P*	.170	.776	.820	.351	.405	.481	.098	.438	.279	.269	1.000	.182	.182

CFS *=* corneal fluorescein staining, LLT *=* lipid layer thickness, NIKBUT *=* noninvasive tear breakup time**,** OSDI *=* Ocular Surface Disease Index, TMH *=* tear meniscus height.

### 3.4. TCM symptom

#### 3.4.1. The relationship between DED indicators and TCM symptoms

We separately analyzed the correlations between 50 TCM symptoms and indicators of DED in different TCM patterns.

Among 56 patients with Qi stagnation and blood stasis pattern, the strongest correlation was found between dry throat and large upper eyelid meibomian gland (*r* = 0.513, *P* = .001). Additionally, patients with waist and knee soreness syndrome showed a thinner lipid layer in the tear film (*r* = −0.467, *P* = .003). Patients with decreased appetite and listlessness syndrome had a lower blink ratio (*r* = −0.442, *P* = .006), patients with muscle and skin nail disorder syndrome had a lower NIBUT grading (*r* = −0.439, *P* = .006), and patients with palpitations and shortness of breath had a lower environmental factor score (*r* = −0.430, *P* = .010).

Among 41 patients with spleen-kidney yang deficiency patterns, patients with limb heaviness had higher visual function scores, showing the strongest correlation (*r* = 0.607, *P* = .004). Additionally, patients with irritability and easy anger had higher ocular symptom scores (*r* = 0.565, *P* = .008). Moreover, patients with dizziness had higher environmental factor scores (*r* = 0.542, *P* = .011), patients with pale lips and nails had higher environmental factor scores (*r* = 0.523, *P* = .015), and patients with cold and painful abdomen had a lower blink ratio (*r* = −0.491, *P* = .020).

Among 40 patients with Qi and Yin deficiency pattern, patients with limb heaviness had higher OSDI visual function scores, showing the strongest correlation (*r* = 0.733, *P* < .001). Additionally, patients with limb heaviness had higher OSDI visual function scores (*r* = 0.714, *P* < .001). Patients with dizziness had higher blink ratios (*r* = 0.573, *P* = .004), patients with headache had higher total scores of CFS (*r* = 0.559, *P* = .008), and patients with nausea and vomiting had lower NIBUT grading (*r* = −0.516, *P* = .014).

## 4. Discussion

The distribution of TCM patterns among patients with DED in this study underscores the multifactorial nature of the condition and highlights the significance of personalized treatment approaches. Consistent with previous studies,^[13–16]^ our findings reveal mixed type DED was the predominant form, particularly among middle-aged females. This underscores the importance of considering both gender and age-related factors in the management of DED. In this study, we showed that mixed-type DED had the highest proportion, with more females than males. The highest incidence rate was observed in the 31 to 40 age group. Among the 11 TCM patterns included in this study, the 3 most common patterns were Qi stagnation and blood stasis, spleen-kidney yang deficiency, and Qi and Yin deficiency. Among patients with mixed type and evaporative DED, the most common TCM patterns were Qi stagnation and blood stasis, spleen-kidney yang deficiency, and Qi and Yin deficiency, respectively. Among patients with the aqueous deficiency type, the most common TCM patterns were Qi stagnation and blood stasis, cold coagulation and blood stasis, and Qi and Yin deficiency, respectively. Qi stagnation and blood stasis were the most common constitutional type among patients with DED, while the aqueous deficiency type of DED was associated with cold coagulation and blood stasis syndrome.

According to TCM, diseases are caused by the interaction of internal and external factors. Internal factors refer to constitutional factors, while external factors refer to pathogens. Disease manifestations are related to the nature of the pathogenic factors and their transformation in the body. We have summarized that the occurrence of any type of DED is related to “dryness.” Pathogenic dryness can be classified into “warm dryness” and “cool dryness” based on the nature of the coexisting pathogenic cold and heat. It can also manifest as “cold transformation” or “heat transformation” depending on individuals’ constitutional differences. Dryness can arise both externally and internally. DED caused by environmental factors, lifestyle habits, dietary habits, eye usage habits, and eye diseases can be attributed to external factors. DED caused by dysfunction of the 5 viscera and 6 bowels or prolonged invasion of pathogenic factors can be attributed to internal factors. Qi stagnation and blood stasis are often caused by emotional distress and liver dysfunction, leading to poor Qi circulation and impaired blood flow. Qi stagnation leads to internal heat and the drying of turbid body fluids. Patients with symptoms of dry throat and mouth show a greater extent of meibomian gland loss, and patients with waist and knee soreness have a thinner layer of tear lipid. We found that Qi stagnation and blood stasis may affect the function of the meibomian gland, decreasing the quality of the lipid layer in tears. Qi stagnation and blood stasis lead to internal dryness, which not only depletes the body fluids but also impairs the nourishment to the tissues and organs. Patients with spleen-kidney yang deficiency experience chronic disease that consumes their spleen and kidney yang, leading to water retention due to yang deficiency. This prevents the vaporization and transformation of body fluids, impairing the nourishment to the eyes and leading to “dryness.” In patients with Qi and Yin deficiency, the lack of Qi weakens the propulsion of Qi and body fluids, leading to poor Qi circulation, impaired blood flow, and abnormal distribution of body fluids. The resulting stagnation and heat induce internal dryness. Yin deficiency promotes the formation of internal heat, further contributing to internal dryness and DED. In the aqueous deficiency type of DED, although the essence is the invasion of pathogenic cold, the internal presence of cold leads to Qi stagnation, and Qi stagnation exacerbates blood stasis, finally resulting in lifelong internal dryness and DED. Therefore, chronic illness gives rise to “dryness,” and “dryness” leads to DED.

Among the various TCM patterns identified before,^[16,17]^ Qi stagnation and blood stasis, spleen-kidney yang deficiency, and Qi and Yin deficiency were the most prevalent patterns in patients with DED. Often associated with emotional stress and sedentary lifestyles, Qi stagnation and blood stasis contribute to microcirculatory impairments in the ocular tissues, exacerbating dry eye symptoms.^[18,19]^ Spleen-kidney yang deficiency, characterized by metabolic dysfunction and fluid imbalance, results in inadequate tear production and lubrication, leading to ocular discomfort.^[20]^ Qi and Yin deficiency can further exacerbate ocular dryness and irritation, reflecting imbalances in vital energy and bodily fluids.^[21]^ These patterns collectively underscore the intricate interplay between physiological systems in the pathogenesis of DED.

Moreover, the association between specific TCM patterns and DED subtypes provides invaluable insights into the mechanisms underlying DED. While Qi stagnation and blood stasis are commonly observed across different subtypes of DED, cold coagulation and blood stasis syndrome are particularly linked to the aqueous deficiency type of DED. This suggests distinct pathophysiological processes, with cold-induced stagnation exacerbating tear film instability and ocular surface damage.

Previous studies have frequently employed the TCM Nine-Constitution Classification for DED constitution research,^[22–24]^ offering valuable clinical references, yet their guidance for practical treatment remains limited. While Chinese herbal medicine and acupuncture demonstrate efficacy for specific DED patterns^[25–27]^, dedicated studies on TCM pattern differentiation for DED are scarce. This study adopts a comprehensive approach integrating Qi-Blood-Yin-Yang pattern differentiation, Zang-Fu pattern differentiation, and Eight Principles pattern differentiation. By analyzing correlations between TCM patterns and DED subtypes, symptoms, and signs, we establish a concise integrated diagnosis-treatment model. This facilitates early identification of pathogenic risks and timely intervention, aligning with the TCM principle of “preventing disease progression in pre-symptomatic stages. “Taking the “Qi-Stagnation and Blood-Stasis” pattern as an example: These patients show higher susceptibility to evaporative DED, with significantly shorter NIBUT and stronger negative correlations with key DED indicators. This implies treatment should prioritize tear film stabilization. Beyond conventional artificial tears (supplementing lipids/proteins), adjuvant therapies, including acupuncture, periocular moxibustion, warm compresses, fumigation, and meibomian gland massage, enhance outcomes by regulating periocular Qi, improving circulation, and promoting lipid excretion. Concurrently, systemic correction of the constitutional imbalance fundamentally resolves Qi-Stagnation and Blood-Stasis pathology, promoting distribution/metabolism of Qi, Blood, and body fluids to reduce DED recurrence.

TCM pattern management emphasizes lifestyle adjustments per the axiom: “Prolonged visual exertion depletes Blood; prolonged inertia impairs Qi.” For such patients, we advise increased physical activity and reduced screen time indoors to mitigate DED risk.In summary, TCM pattern-based integrated diagnosis and treatment delivers holistic, personalized care that effectively alleviates DED symptoms and improves quality of life.

This study investigated the distribution of TCM patterns in DED and observed the distribution of TCM patterns in different types of DED. Certain patterns were identified by combining the patterns with symptoms and signs, providing direction for future research on TCM in DED. Despite the insights provided by our study, several limitations need to be considered. The small sample size, single-center design, and limited participant numbers in certain groups may impact the accuracy and generalizability of our findings, emphasizing the need for larger, multicenter studies with more diverse populations. Additionally, the subjective nature of TCM pattern identification and the lack of standardized diagnostic criteria may introduce variability in pattern differentiation, so future studies will focus on validating the questionnaire through reliability and validity analysis. Future research efforts should focus on refining diagnostic methods and exploring the potential synergies between TCM and conventional treatments for managing DED. This study can serve as a starting point for future well-designed prospective studies to establish a standardized research system for TCM in DED. We need to deepen and refine our research to find more options for treating dry eye, including herbal formulations and acupuncture. These interventions target specific TCM patterns, addressing both the underlying imbalances and symptomatic relief.

## 5. Conclusion

Our study provides a deeper insight into TCM patterns in DED and their clinical implications. By elucidating the complex interplay between TCM patterns and DED subtypes, we pave the way for personalized and integrative approaches to DED. It provides ideas for establishing a concise TCM prevention, diagnosis and treatment pattern for dry eye.

## Acknowledgments

We sincerely appreciate Director Qiu Lixin from the Department of Traditional Chinese Medicine, Beijing Tongren Hospital, for his valuable guidance and expertise in Traditional Chinese Medicine theory. The authors would like to express their gratitude to EditSprings (https://www.editsprings.cn) for the expert linguistic services provided.

## Author contributions

**Conceptualization:** Jing Yu, Lei Tian, Lixin Qiu, Ying Jie.

**Methodology:** Jing Yu.

**Supervision:** Jing Yu, Lei Tian, Lixin Qiu, Ying Jie.

**Writing – review & editing:** Jing Yu.

**Formal analysis:** Yihan Guo.

**Writing – original draft:** Yihan Guo.

**Data curation:** Xiaoniao Chen, Kai Cao.

**Validation:** Lei Zhu, Mengnan Zhao.
